# The emerging roles of ferroptosis in cells of the central nervous system

**DOI:** 10.3389/fnins.2022.1032140

**Published:** 2022-12-14

**Authors:** Yuyao Li, Dongqiong Xiao, Xiaodong Wang

**Affiliations:** ^1^Department of Obstetrics and Gynecology, West China Second University Hospital, Sichuan University, Chengdu, China; ^2^West China School of Medicine, Sichuan University, Chengdu, Sichuan, China; ^3^Department of Emergency, West China Second University Hospital, Sichuan University, Chengdu, China; ^4^Key Laboratory of Birth Defects and Related Diseases of Women and Children, Ministry of Education, Department of Emergency, West China Second University Hospital, Sichuan University, Chengdu, China

**Keywords:** astrocyte, ferroptosis, oligodendrocyte, microglia, pericyte

## Abstract

Ferroptosis is morphologically characterized by shrunken mitochondria and biochemically characterized by iron overload, lipid peroxidation and lipid reactive oxygen species (ROS) accumulation; these phenomena are suppressed by iron chelation, genetic inhibition of cellular iron uptake, and intervention on other pathways such as lipid metabolism. The induction of ferroptosis may be related to pathological cellular conditions in the central nervous system (CNS); thus, ferroptosis may cause disability via CNS damage. Here, we review the role of ferroptosis in the main cells of the CNS, including glial cells, neurons, and pericytes; in various diseases of the CNS; and in the interaction of glia and neurons in CNS diseases. Some small molecules and traditional Chinese drugs which inhibit ferroptosis in cells of the CNS are shown as potential therapeutic strategies for neurological diseases.

## Introduction

### Overview of the history of ferroptosis

Ferroptotic death, which is also known as ferroptosis, is a nonapoptotic form of cell death that was first identified by Dixon et al. (2012). Dixon et al. (2012) first observed that erastin-induced cancer cell death, which they called ferroptosis, involves a unique combination of morphological, biochemical, and genetic characteristics that are distinct from those of apoptosis, necrosis, and autophagy. These authors found that the distinct morphological changes that occur during ferroptosis include mitochondria that appear to be smaller than normal and have increased membrane density on transmission electron microscopy. Distinctive morphological changes occur with the following three forms of cell death: apoptosis is characterized by chromatin condensation and margination, necrosis is characterized by cytoplasmic and organellar swelling and plasma membrane rupture and autophagy is characterized by the formation of double-membrane-enclosed vesicles. Ferroptosis is characterized by the accumulation of iron and lipid ROS and is suppressed by iron chelation or genetic inhibition of cellular iron uptake (Dixon et al., 2012; Stockwell et al., 2017). Ferroptosis occurs in both human and mouse cells; here, we review the roles of ferroptosis in cells of the CNS, including neurons, astrocytes, oligodendrocytes, microglia and pericytes.

### Definition of ferroptosis

Ferroptosis is a unique iron-dependent form of nonapoptotic cell death that is characterized by iron accumulation, lethal levels of lipid peroxidation and depletion of plasma membrane unsaturated fatty acids (Dixon et al., 2012; Weiland et al., 2019). As observed in electron micrographs, ferroptotic cells exhibit small mitochondria with condensed mitochondrial membranes, a decreased number of mitochondrial cristae, and ruptured outer mitochondrial membranes (Stockwell et al., 2017; Weiland et al., 2019).

**FIGURE 1:**
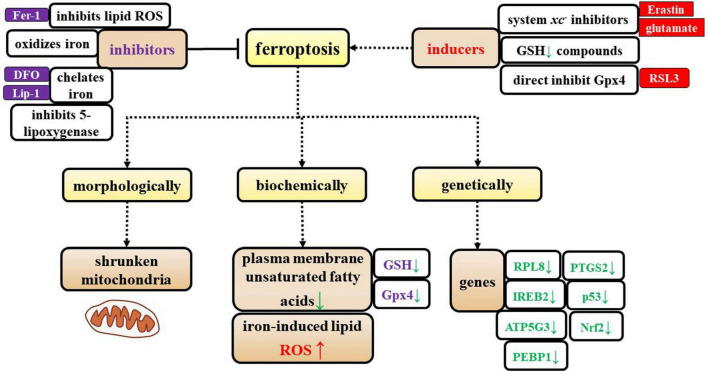
Characterization of ferroptosis. The specific characteristics of ferroptosis are shown from three perspectives: morphological, biochemical, and genetic alterations. Additionally, the inducers and inhibitors of ferroptosis are shown in this figure.

Ferroptosis can be suppressed by iron chelators, lipophilic antioxidants, and inhibitors of lipid peroxidation and 5-lipoxygenase. Ferroptosis can be induced by the accumulation of glutamate, iron, or PUFA-phospholipids, or by the inhibition of system *Xc*^–^, GSH depleting compounds, and glutathione peroxidase 4 (Gpx4) (Figure 1).

Additionally, Dixon et al. (2012) identified genes that specifically regulate ferroptosis, including iron response element binding protein 2 (IREB2), which encodes a master regulator of iron metabolism, and the IREB2 negative regulator f-box and leucine-rich repeat protein (FBXL5); these two regulatory genes cause reciprocal changes in the expression of genes known to participate in iron uptake, metabolism, and storage, including the TFRC, ferritin heavy chain 1 (FTH1), and ferritin light chain (FTL), as well as genes that are related to erastin sensitivity. Dixon et al. (2012) identified specific roles in erastin-induced ferroptosis for most of the known genes that encode mitochondrial proteins, including ribosomal protein L8 (RPL8), iron response element binding protein 2 (IREB2), ATP synthase F0 complex subunit C3 (ATP5G3), ER membrane protein complex subunit 2 (TTC35/EMC2), citrate synthase, and acyl-CoA synthetase family member 2 (ACSF2). Later, other researchers showed that prostaglandin-endoperoxide synthase 2 (PTGS2), nuclear factor erythroid 2-related factor 2 (Nrf2), p53, and phosphatidylethanolamine-binding protein 1 (PEBP1) participate in ferroptosis (Abdalkader et al., 2018; Gou et al., 2020; Liu et al., 2020; Jiao et al., 2021; Li X. et al., 2021) (Figure 1).

### Glutathione (GSH)

Glutathione, which is an essential intracellular antioxidant, is synthesized from cysteine, glutamate, and glycine in two steps that are catalyzed by the adenosine-5-triphosphate (ATP) -dependent cytosolic enzymes GCL and GSS (Stockwell et al., 2017). Cysteine availability limits the GSH synthesis rate (Stockwell et al., 2017). GSH is a cofactor of Gpx4 that is necessary for eliminating lipid peroxides. Inhibiting the glutamate/cysteine antiporter system *Xc*^–^, which is a key antioxidant defense system in the CNS, can induce ferroptosis. Pharmacological analysis revealed that ferroptosis is a major factor that contributes to glutamate-initiated oligodendrocyte death (Stockwell et al., 2017).

Glutathione depletion caused by erastin is sufficient to induce ferroptosis. The significant depletion of GSH/GSSG is consistent with the fact that erastin induces ROS production, causing oxidative cell death (Yang et al., 2014).

### Gpx4

In the brain, GPX enzymes, which act as antioxidants, are expressed in neurons and glial cells (Cardoso et al., 2017). Gpx4 the only member of the GPX family, is synthesized endogenously in the brain, it can reduce lipid peroxides and ROS production, and it was recently recognized as the major inhibitor of ferroptosis (Chen et al., 2015; Cardoso et al., 2017).

### Lipid metabolism

**FIGURE 2:**
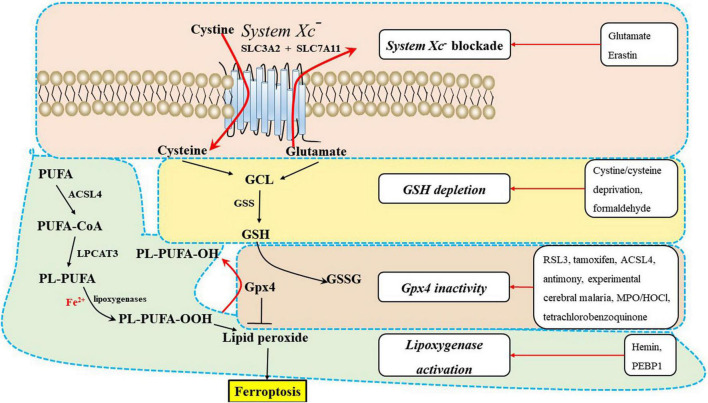
The underlying mechanisms of ferroptosis regulation in the central nervous system (CNS). Ferroptosis inducers are shown in the box, including system *Xc*^–^ blockade, GSH depletion, Gpx4 inactivity, and lipoxygenase activation. ACSL4, acyl-CoA synthetase long-chain family member 4; GPX4, glutathione peroxidase 4; GSH, glutathione; GSS, glutathione synthetase; LPCAT3, lysophosphatidylcholine acyltransferase 3; PEBP1, phosphatidylethanolamine-binding protein 1; PUFA-CoA, polyunsaturated fatty acid; PL-PUFA, polyunsaturated fatty acid (PUFA)-phospholipids; PL-PUFA-OOH, phospholipid hydroperoxide; MPO/HOCl, myeloperoxidase/hypochlorous acid.

Polyunsaturated fatty acids, which contain bis-allylic hydrogen atoms, are susceptible to lipid peroxidation and are essential for ferroptosis (Abdalkader et al., 2018; Weiland et al., 2019). The degree of ferroptosis is measured by the amount of lipid peroxidation, which is caused by the abundance and localization of PUFAs. Free PUFAs, which are substrates for the synthesis of lipid signaling mediators, must be esterified into membrane phospholipids and initially undergo oxidation to participate in ferroptosis related signaling pathways (Jiang et al., 2021). Ferroptosis is induced or suppressed by regulatory enzymes that are involved in the biosynthesis of PUFA-containing membrane phospholipids, such as acyl-CoA synthetase long-chain family member 4 (ACSL4) and lysophosphatidylcholine acyltransferase 3 (LPCAT3). Neuronal plasma membranes are rich in PUFAs, which are prone to free radical attack and peroxidation of unsaturated carbon-carbon bonds. Enzymatic effectors known as lipoxygenases (LOXs) can mediate lipid peroxidation and result in ferroptosis, and free PUFAs are preferred substrates of LOXs (Figure 2) (Weiland et al., 2019).

### Amino acid metabolism

**FIGURE 3:**
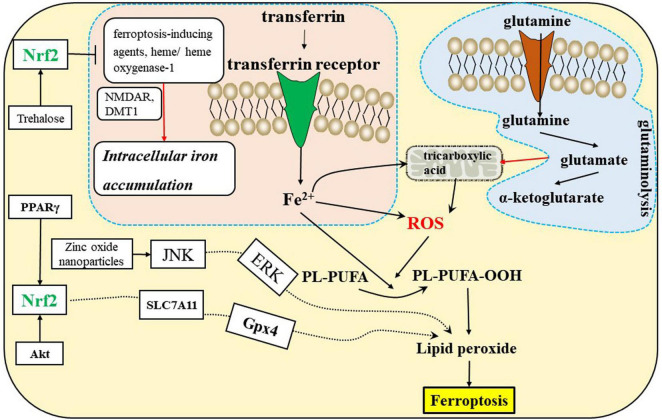
Ferroptosis is related to the iron, amino acid metabolism, and Nrf2 signaling pathways in the CNS. DMT1, divalent metal transporter 1; NMDAR, *N*-methyl-D-aspartate receptors; Nrf2, nuclear factor erythroid 2-related factor 2; PPARγ, peroxisome proliferator-activated receptor γ.

Amino acid metabolism is tightly linked to ferroptosis (Stockwell et al., 2017). First, GSH biosynthesis was limited by cysteine availability and ferroptosis was induced by cystine starvation. Second, glutamate induces ferroptosis via inhibiting system *Xc*^–^. Third, glutamine is enriched in human brain tissues, and its degradation (via glutaminolysis) provides fuel for the tricarboxylic acid (TCA) cycle (Figure 3) and building blocks for lipid biosynthesis. α-ketoglutarate (αKG), a product of glutaminolysis, is required for ferroptosis (Stockwell et al., 2017; Jiang et al., 2021).

### Iron metabolism

Iron is required for lipid peroxide accumulation, which is the execution of ferroptosis (Stockwell et al., 2017; Jiang et al., 2021). Ferroptosis sensitivity is affected by iron import, export, storage, and turnover. Transferrin and TFRC promote ferroptosis by importing iron from the extracellular environment into the cell. Furthermore, heme oxgenase-1 is associated with ferroptosis by degrading heme liberating iron (Jiang et al., 2021).

## Inducers and inhibitors of ferroptosis

### Ferroptosis induction

There are many ways to induce ferroptosis, including system *Xc*^–^ blockade [glutamate, erastin, Ras selective lethal 3 (RSL3)], reducing GSH components, directly inhibiting the Gpx4 enzyme ACSL4 (Cui et al., 2021a), tetrachlorobenzoquinone (Liu et al., 2021), RSL3, tamoxifen, antimony (Yu et al., 2022), and zinc oxide nanoparticles (Qin et al., 2020), substantially increasing lipid peroxidation (FEBP1).

### Ferroptosis inhibition

There are many ways to inhibit ferroptosis, including reducing lipid ROS production {ferrostatin-1 [Fer-1 (Chen et al., 2019)], ADA-409-052}, blocking lipid peroxidation {vitamin E, coenzyme Q10 (CoQ10), idebenone (Avcı et al., 2021), liproxstatin-1 [Lip-1 (Cao et al., 2021)]}, chelating iron (DFO and DFP), or iron oxide, and inhibiting 5-lipoxygenase.

## Ferroptosis in cells of the CNS

### Cell to cell interaction

There are many types of cells in the CNS, including glial cells, neurons, and pericytes (Yao et al., 2012; Czepiel et al., 2015; Ji et al., 2019; Urbán et al., 2019). Oligodendrocytes, astrocytes, and microglia are the glial cells abundant in CNS. Neurons interact with astrocytes, oligodendrocytes, and microglia to maintain homeostasis of the brain.

In glial cells, including oligodendrocytes, astrocytes, and microglia, crosstalk during myelination is complicated. Quiescent astrocytes and steady-state microglia may regulate (promote or suppress) OPC differentiation and myelination via a specific program in the CNS.

The OPCs are more susceptible to exposure to extracellular free iron, which leads to increased lipid peroxidation, arrested oligodendrocyte differentiation, and cell death—all key hallmarks of ferroptosis (Back et al., 2002; Nobuta et al., 2019). Reactive astrocytes and activated microglia can promote or inhibit the process of remyelination or increase oligodendrocyte ferroptosis (Domingues et al., 2016). Additionally, in rats, OPCs are more susceptible to cysteine depletion than primary oligodendrocytes (Hoshino et al., 2020).

Microglial iron status may be important for oligodendrocyte survival. Iron overload promotes microglial H-ferritin release, leading to increased oligodendrocyte survival, but knocking down H-ferritin in iron-loaded microglia leads to decreased oligodendrocyte survival (Feng et al., 2021).

The antioxidant transcription factor Nrf2 regulates hundreds of genes present in brain cells, many of which are either directly or indirectly involved in modulating ferroptosis, including metabolism of GSH, lipids and iron, and mitochondrial function (Abdalkader et al., 2018; Ishii et al., 2019; Jiang et al., 2021). GSH-related genes regulated by Nrf2, including solute carrier family 7 member 11(SLC7A11), Gpx4, and GSS (Figure 2), exist in oligodendrocytes, astrocytes, and neurons. Iron-related genes regulated by Nrf2 include ferritin heavy chain 1 (FTH1), transferrin receptor 1 (TfR-1), and heme oxygenase-1, which also exist in brain cells. The NADPH-regeneration related genes regulated by Nrf2 are glucose-6-phosphate dehydrogenase (G6PD) and phosphogluconate dehydrogenase (PGD). In addition, Nrf2 regulates lipid synthesis via the ligand-mediated transcription factor PPARγ, which is enriched in oligodendrocytes, neurons, astrocytes and other brain cells. Accordingly, Nrf2 indirectly regulates lipids whose abundance contributes to sensitivity to ferroptosis. In conclusion, we speculate that inadequate activation of Nrf2 may induce ferroptosis of brain cells and disrupt the interactions of brain cells, which can lead to CNS diseases (Abdalkader et al., 2018; Weiland et al., 2019). There is mounting evidence that dysregulation of glial-neuronal interactions can lead to CNS diseases.

Astrocyte-neuron interactions protect neurons from ferroptosis. Brain-derived neurotrophic factor (BDNF) in astrocytes effectively activates Nrf2, an inhibitor of ferroptosis, during the resting phase and regulates the metabolic cooperation between astrocytes and neurons. Circadian increases in the levels of circulating glucocorticoids may further facilitate material transfer from astrocytes to neurons by stimulating the pannexin 1 channel-P2 × 7R signaling axis in astrocytes at the beginning of the active phase (Ishii et al., 2019). Some studies have reported that astrocytes protect neurons from ferroptosis because astrocytes have a high capacity to store iron and prevent neuronal iron overload (Codazzi et al., 2015). Additionally, inadequate Nrf2 activation in astrocytes and dysregulation of astrocytic neuronal interactions may induce neuronal ferroptosis (Cui et al., 2016; Ishii et al., 2019). Aquaporin 4 (AQP4), which is a water channel protein located in the astrocytic endfeet, is related to BBB integrity, the disruption of which is an important cause of SAH. APQ4 overexpression in astrocytes effectively reduces transferrin infiltration and neuronal ferroptosis after SAH. Liu et al. (2022) indicated that AQP4 overexpression may inhibit transferrin infiltration into the brain parenchyma in the lymphatic system. Treatment with the ferroptosis inhibitor SRS 16-86 enhances functional recovery after SCI through the upregulation of the anti-ferroptosis factors Gpx4, GSH, and SLC7A11, and the downregulation of 4-HNE (lipid peroxidation marker). SRS 16-86 treatment alleviates astrogliosis and enhances neuronal survival after SCI (Zhang et al., 2019). Pantothenate kinase-associated neurodegeneration (PKAN) is caused by mutations in the *PKAN* gene, which encodes the mitochondrial enzyme *PKAN;* this protein catalyzes the first reaction of the CoA biosynthetic axis. *PKAN*-mutant astrocytes, which demonstrate signs of ferroptosis, are prone to acquire a stellate phenotype and neurotoxicity, indicating alterations in iron metabolism, mitochondrial morphology, respiratory activity, and oxidative status (Santambrogio et al., 2022).

The interaction of oligodendrocytes and neurons, including oligodendrocyte development and myelin membrane sheath generation, plays an essential role during the development of the nervous system (Domingues et al., 2016). Oligodendrocyte ferroptosis may lead to oligodendrocyte maturation arrest, myelin sheath damage and PVL, and myelin sheath damage can stimulate microglial activation and neuronal damage. Additionally, activated microglia contact damaged axons and are thus correlated with axonal damage (Czepiel et al., 2015; Domingues et al., 2016; Kapralov et al., 2020).

Microglial iron status may be important for neuron survival. Microglial activation induces iron overload in the motor cortex after SCI, triggering ferroptosis of motor neurons and impeding the recovery of motor function (Feng et al., 2021). Microglia interact with neurons and astrocytes to participate in ferroptosis under pathological conditions. For example, a small molecule with an arylthiazine backbone (ADA-409-052) inhibits tert-butylhydroperoxide (t-BHP)-induced lipid peroxidation, thus suppressing the activation of proinflammatory BV2 microglia, inhibiting neuronal ferroptosis, and efficiently reducing the infarct volume, oedema and proinflammatory gene expression in a thromboembolic stroke mouse model (Keuters et al., 2021). The abundance of divalent metal transporter 1 (DMT1) on the surface of astrocytes, microglia and neurons is increased under neuroinflammatory conditions, and these cells are susceptible to ferroptosis due to iron overload caused by high levels of non-transferrin-bound iron. Inactivation of DMT1 by ferroptosis inhibitors such as CoQ10, DFO, DFP, deferasirox (DFX), and *N*-acetylcysteine may play a protective role under pathological conditions (Morris et al., 2018). Hypoxia-inducible factor 1-alpha (HIF-1α) inhibits ferroptosis by downregulating ACSL4-dependent lipid metabolism. Moreover, knockdown of ACSL4 inhibited proinflammatory cytokine production in microglia. Therefore, ACSL4, as an inducer of neuronal ferroptosis and neuroinflammation, may be a potential therapeutic target in ischemic stroke (Cui et al., 2021a). Homeostasis of the CNS depends on crosstalk among astrocytes, oligodendrocytes, microglia, and neurons. Here, we review ferroptosis in these cells and the roles of ferroptosis in common CNS disease models.

### Ferroptosis of neurons

Gpx4, an inhibitor of ferroptosis, is essential for motor neuron survival *in vivo* (Schriever et al., 2017). Gpx4 ablation-induced neuronal degeneration in the cerebral cortex exhibits features of ferroptosis, including lipid peroxidation and mitochondrial dysfunction (Chen et al., 2015). Gpx4 deletion from dopaminergic neurons triggers anxiety behavior, and DJ-1 codeletion is accompanied by a decrease in spontaneous locomotor function (Schriever et al., 2017). Moracin N, which is a ferroptosis inhibitor, is involved in antioxidant defense and GSH biosynthesis (Wen et al., 2021). Nrf2, which is an inhibitor of neuronal ferroptosis that reduces iron-induced oxidative stress, alleviates the decrease in GSH levels by increasing the expression of genes related to GSH synthesis, such as SLC7A11 and GCL (Liu et al., 2020).

Tetrachlorobenzoquinone triggers neuronal ferroptosis, as shown by observations of lipid peroxidation and shrunken mitochondria via Gpx4 ablation and iron accumulation (Liu et al., 2021). General anesthesia induced iron overload is activated by *N*-methyl-D-aspartate receptor (NMDAR)-RASD1 signaling via divalent metal transporter 1 (DMT1) (Wu J. et al., 2020). Antimony induces ferroptosis through Gpx4 degradation by chaperone-mediated autophagy (CMA) (Yu et al., 2022). Zinc oxide nanoparticles selectively induce ferroptosis by activating the c-Jun NH2-terminal kinases (JNK) pathway, and the inhibitor SP600125 reverses lipid peroxidation (Qin et al., 2020). Formaldehyde induces ferroptosis, which is accompanied by peroxidative stress; GSH downregulation; MDA, 4-hydroxynonenal (4-HNE), ROS and iron upregulation; and enhanced expression of ferroptosis related genes, including PTGS2, glutaminase 2 (GLS2), solute carrier family 1 member 5 (SLC1A5), and solute carrier family 38 member 1 (SLC38A1) (Li X. et al., 2021). Arsenite is an inducer of neuronal ferroptosis, hallmarks of ferroptosis that are associated with arsenite toxicity include ROS accumulation, increased lipid peroxidation, Fe^2+^ homeostasis disruption, GSH and adenosine triphosphate depletion, system *Xc*^–^ inhibition, activation of MAPK and mitochondrial voltage-dependent anion channel pathways, and increased endoplasmic reticulum stress (Tang et al., 2018). Tert-butylhydroperoxide (t-BHP) is another inducer of ferroptosis; its effects include JNK1/2 and extracellular regulated protein kinases (ERK)1/2 activation upstream of ferroptosis and mitochondrial dysfunction (Wu et al., 2018).

### Ferroptosis of microglia

Microglia are scavenger cells that account for approximately 10% of all glial cells in the CNS; microglia are involved in phagocytosis, inflammatory responses, cytoplasmic migration and secretion of growth factors, and immune responses in the CNS. Microglia in the M0 (resting) state have rod-shaped cell bodies under normal conditions. The activation of microglia after CNS injury causes their polarization into either the M1 (proinflammatory) phenotype or the M2 (anti-inflammatory) phenotype, and these cells may produce pro- and anti-inflammatory cytokines or chemokines, which exert neurotoxic or neuroprotective roles under pathological conditions (Yu et al., 2020).

Fer-1, a ferroptosis inhibitor, accelerates microglial M2 polarization, enhances microglial phagocytosis, and prevents inflammation in Hb-treated organotypic hippocampal slices (Huang et al., 2022). Liproxstatin-1, a ferroptosis inhibitor, decreases microglial activation and IL-6, IL-1β, and TNF-α secretion (Cao et al., 2021). Microglial activation may trigger ferroptosis. RSL3 induces Nrf2 protein expression, inhibiting the recruitment of RNA polymerase II to the transcription start sites of proinflammatory cytokine genes to suppress cytokine transcription and protect cells from ferroptosis (Cui et al., 2021b).

Exposure of microglial BV2 cells to nitrogen-doped graphene quantum dots (N-GQDS) can induce microglial ferroptosis, which is characterized by lipid peroxidation, GSH depletion, excessive ROS production, and iron accumulation in the mitochondria (Wu T. et al., 2020). Ferroptosis is common in the proinflammatory environment. Kapralov et al. (2020) found that iNOS/NO(•)-mediated enrichment of activated M1 macrophages/microglia modulates susceptibility to ferroptosis.

### Ferroptosis of oligodendrocytes

The main function of neurons, astrocytes, microglia and oligodendrocytes in the CNS is to form myelin sheaths to enclose axons, thus playing a role in neurotransmission in the CNS. Oligodendrocytes are the main cells that form myelin sheaths; oligodendrocytes differentiate from NSCs through specific signaling pathways and genetic programs, such as those involving epigenetic regulators (microRNAs and long non-coding RNAs), that regulate the differentiation of OPCs into oligodendrocytes (Xiao et al., 2018). OPCs have the ability to proliferate and migrate. After proliferating, OPCs can differentiate into oligodendrocytes and express specific markers, such as myelin basic protein, myelin associated glycoprotein and PLP (Back and Rivkees, 2004; Segovia et al., 2008).

In the adult brain, OPCs and oligodendrocytes contain up to 20 times more iron than astrocytes. One of the main reasons for the enrichment of iron levels in oligodendrocytes is that iron is a cofactor that is necessary for myelin synthesis (Stockwell et al., 2017). Pretreatment of mutant O4^+^ OPCs with the iron chelators DFO and deferiprone (DFP) reduces lipid ROS^+^ cells and enhances oligodendrocyte survival, differentiation, and new myelin formation in the brains of mice *in vivo* (Nobuta et al., 2019). Inhibitors of ferroptosis, such as Lip-1, Fer-1, and Gpx4, localize to the nuclei of CAII^+^ oligodendrocytes, reduce mitochondrial lipid ROS production and lipid peroxidation, and promote OPC maturation and myelination (Fan et al., 2021).

However, iron overload often leads to ferroptosis. Iron overload induces oxidative stress injury, and multiple studies have reported that oligodendrocytes are sensitive to oxidative stress injury (Hu et al., 2019; Nobuta et al., 2019; Hoshino et al., 2020). Additionally, inhibiting system *Xc*^–^-mediated cystine uptake by erastin, SAS, or glutamate may be sufficient to initiate iron-dependent ferroptosis. Glutamate triggers oligodendrocyte death via a mechanism that is dependent on excitotoxicity, opening of the mitochondrial permeability transition pore, enhanced ROS production, and augmented lipid peroxidation, and this form of cell death is caused by persistent activation of ionotropic glutamate receptors, blockade of cysteine uptake, and/or oxidative stress injury induced by system *Xc*^–^. These results indicated that ferroptosis induces oligodendrocyte maturation arrest and inhibits myelination or demyelination (Hoshino et al., 2020; Jhelum et al., 2020; Ma et al., 2020).

### Ferroptosis of astrocytes

Astrocytes are glial cells that originate from NSCs in the subventricular zone, and astrocytes interact with neurons, oligodendrocytes, microglia, and pericytes to maintain homeostasis of the brain, including molecular homeostasis (homeostasis of ions, such as K^+^, Na^+^, Ca^2+^, and homeostasis of neurotransmitters, such as glutamate, gamma-aminobutyric acid, adenosine, monoamines), cellular and network homeostasis (such as neurogenesis, neuron development, synaptic maintenance and elimination, and synaptic plasticity), metabolic homeostasis (glycol-gene synthesis and storage), organ homeostasis (BBB regulation), and systemic homeostasis (chemosensing of oxygen and CO_2_) (Sofroniew, 2020). Additionally, astrocytes also express the SLC7A11, which is important for the accumulation of cysteine that is required for production of GSH, which participates in ferroptosis. Three types of aquaporins, namely, AQP1, AQP4, and AQP9, have been identified in astrocytes, and AQP4 in particular is abundant in these cells. Deletion of AQP4 results in decrease in astrocyte water permeability, deficient K^+^ buffering, and deficits in synaptic plasticity, while upregulation of AQP4 leads to water absorption by astrocytes. Additionally, pannexin-1 and P2 × 7 receptor transcripts were identified in astrocytes, and these molecules may participate in astrocyte ferroptosis (Sofroniew, 2020).

Ferroptosis in astrocytes (identified by elevated ROS levels and downregulated GSH and Gpx4 levels) may be induced by the angiotensin II (Ang II)-activated Nrf2/heme oxygenase-1 signaling pathway and may be greatly suppressed by treatment with Fer-1 (Li S. et al., 2021).

Defective *pantothenate kinase-2 (PKAN)* in astrocytes indicates iron deposition/overload and changes in mitochondrial morphology, respiratory activity, and oxidative status (Santambrogio et al., 2022).

### Ferroptosis of pericytes

Pericytes, which specifically express platelet derived growth factor receptor β (PDGFRβ), are a vital part of the neurovascular unit. Pericyte deficiency may be related to increased permeability of the BBB and has been considered to be a factor that initiates disease in some models, such as models of abnormal BBB leakage, oedema, microaneurysm formation, and ischemia (Su et al., 2019). The number of pericytes decreases in APP/PS1 mice. Aβ1–40 induced ferroptosis occurs in pericytes with increased Fe^2+^ levels, increased lipid ROS levels, and GSH depletion. Aβ1–40 disrupts the BBB by inducing pericyte mitophagy-dependent ferroptosis via the cluster 36 (CD36)/PINK1/Parkin axis (Li et al., 2022).

## Ferroptosis in brain diseases

Ferroptosis has been shown to play an important role in the progression of numerous brain diseases, including PD (Hou et al., 2019; Avcı et al., 2021), AD (Hambright et al., 2017), ALS (Devos et al., 2020), cerebral ischemia (Lan et al., 2020; Cui et al., 2021a), ICH (Chen et al., 2019; Bai et al., 2020), SAH (Qu et al., 2022), PMD (Nobuta et al., 2019), PVL, MS, EAE (Hu et al., 2019), SCI, TBI (Rui et al., 2021), and depression (Jiao et al., 2021). System *Xc*^–^ blockade, GSH depletion, Gpx4 inactivity, lipoxygenase activation (Figure 2), intracellular iron accumulation (Figure 3), and some related signaling pathways (Jiang et al., 2021) (Figure 3) are common ferroptotic mechanisms in CNS diseases. Inhibitors of ferroptosis play neuroprotective roles in CNS diseases. Some small molecules and traditional Chinese drugs that inhibit ferroptosis in CNS cells have been shown to be underlying therapeutic strategies for CNS diseases (Table 1).

**TABLE 1:** The roles of ferroptosis in different central nervous system (CNS) cell types and brain diseases.

CNS cell type	Important molecular alterations	Model	Mechanism	References
**Neurons**				
	Gpx4↓	ALS	Tamoxifen eliminates Gpx4	Wang T. et al., 2022
	Gpx4↓	ALS	MPO/HOCl pathway inhibits Gpx4 and NQO1	Peng et al., 2022
	pPFFs↑	PD	Liproxstatin-1 inhibits ferroptosis	Guiney et al., 2020
	NADPH↑	PD	Idebenone down regulates lipid peroxidation; Gpx4↑	Avcı et al., 2021
	Gpx4↓	PD	Paeoniflorin → Akt/Nef2/Gpx4	Wang L. et al., 2022
	Gpx4↓	Cerebral ischemia	Carvacrol → Gpx4↑	Guan et al., 2019
	Gpx4↓	HIBD	Melatonin → Akt/Nef2/Gpx4	Gou et al., 2020
	Gpx4↓	Cerebral ischemia	Naotaifang → Tfr1/DMT1 and SCL7A11/Gpx4	Lan et al., 2020
	Gpx4↓	ICH	PPARγ → Nrf2/Gpx4	Duan et al., 2022
	Gpx4↓, Nrf2, FTH1 and SLC7A11↓	ICH	Crocin → Nrf2, Gpx4, FTH1 and SLC7A11	Wang F. et al., 2022
	MIB2↑	Postoperative cognitive dysfunction	Sevoflurane → MIB2↑, triggering ferroptosis	Zhao et al., 2021
	Gpx4↓ TfR1 and ACSL4↑	Experimental cerebral malaria	CD8(+) T cells induce ferroptosis	Liang et al., 2022
	Gpx4↓ Transferrin and MDA↑	SAE	NA	Xie et al., 2022
	12/15-LOX↑	Posttraumatic epileptic seizure	Baicalein inhibits 12/15-LOX	Li Q. et al., 2019
	IRP-2, TfR1↑	Central presbycusis	Increased iron entry into cells	Chen et al., 2020
	ROS↑	SCI	ROS inhibitors and ferroptosis reduce iron overload Trehalose → Nrf2/heme oxygenase-1	Feng et al., 2021; Gong et al., 2022
	NA	TBI	Melatonin depends on MT2 to inhibit ferroptosis	Rui et al., 2021
	NA	Type 1 diabetes related cognitive dysfunction	SLC40A1	Hao et al., 2021
**Oligodendrocytes**				
	Mutations in proteolipid protein 1 (PLP1)↓	Pelizaeus–Merzbacher disease (PMD)	The iron chelator deferiprone inhibits ferroptosis and promote myelination	Nobuta et al., 2019
	Gpx4↓	MS and EAE	Augmented lipid peroxidation products and reduced polyunsaturated fatty acid (PUFA) levels	Hu et al., 2019
	Iron release↑ from oligodendrocytes or hemoglobin breakdown products	MS	Hemin-induced demyelination and axonal loss	Baldacchino et al., 2022
	Cuprizone↑	MS	Activation of ferroptosis induces oligodendrocyte loss and demyelination	Jhelum et al., 2020
	Ferroptosis inhibited by liproxstatin-1	RSL-3 induced oligodendrocyte model of ferroptosis	SLC7A11/glutathione/Gpx4 ACSL4↓	Fan et al., 2021
	IREB2 and PTGS2↓	TBI and SCI	Ferrostatin-1 downregulates the ferroptosis related genes IREB2 and PTGS2	Ge et al., 2021
	Gpx4, GSH and system *xc*^–^-mediated cystine transporter↑. Lipid peroxidation (marker 4HNE)↓	SCI	SRS 16-86 inhibits ferroptosis by upregulating Gpx4, GSH and system *xc*^–^-mediated cystine transporter, and downregulating lipid peroxidation (marker 4HNE)	Zhang et al., 2019
	Sodium selenite → specificity protein 1↑ and Gpx4↑	SCI	Sodium selenite inhibit ferroptosis by specificity protein 1 and Gpx4	Chen et al., 2022
	Gpx4↓	IVH-induced WMI	Hemin-induced OPC death/Gpx4/Lipid peroxide accumulation	Shen et al., 2022
**Microglia**				
	Heme oxygenase-1↑	Aging and age-related neurodegenerative diseases	Neurotoxic ferric iron deposits, iron accumulation	Fernández-Mendívil et al., 2021
	Compound with arylthiazine backbone (ADA-409-052)	Thromboembolic stroke	Inhibits tert-butyl hydroperoxide (TBHP)/GSH/Gpx4/lipid peroxidation	Keuters et al., 2021
	DJ-1↑	NA	Iron overload/lipid peroxidation	Morris et al., 2018
	iNOS↓	Brain tumor	Inducible nitric oxide synthase (iNOS)/NO(•)-enrichment of activated M1 macrophages/microglia → ferroptosis	Kapralov et al., 2020
	ALOX15↑	SAH	Associated with Gpx4/GSH↓	Gao et al., 2022
**Astrocyte**				
	Gpx4↓	Depressive-like and anxiety-like behaviors	Edaravone (EDA) exerts an anti-depressant effect via Sirt1/Nrf2/heme oxygenase-1/Gpx4. Xiaoyaosan → PEBP1 inhibits Gpx4.	Jiao et al., 2021; Dang et al., 2022
	ACSL4↓	Neuropathic pain	Ferrostatin-1 → Gpx4↑ and ACSL4↓	Wang et al., 2021
	Transferrin infiltration↑	SAH	AQP4↑ → inhibits transferrin infiltration into the brain parenchyma in the lymphatic system, inhibits ferroptosis	Liu et al., 2022
	NOX4↑ 4-hydroxynonenal (4-HNE) and malondialdehyde (MDA)↑	AD	NOX4 → ferroptosis of astrocytes → lipid peroxidation → impairment of mitochondrial metabolism	Park et al., 2021

↑, upregulation; ↓, downregulation; NA, not available.

### Potential therapeutic targets for Parkinson’s disease (PD)

Parkinson’s disease is characterized by the death of neurons in the substantia nigra pars compacta (SNpc), which leads to motor disability. Researchers have found that neuronal ferroptosis may be related to PD. Pharmacological inhibition of neuronal ferroptosis may serve as a therapeutic strategy for treating PD. For example, preformed α-synuclein fibrils induce ferroptosis in PD, and this effect can be reversed by high doses of the ferroptosis inhibitor liproxstatin-1 (Guiney et al., 2020).

Idebenone, which is an analog of CoQ10, inhibits striatal NADPH dehydrogenase[quinone]-1 reduction, decreases the striatal levels of lipid peroxidation, and upregulates Gpx4, thus exerting protective effects in PD (Avcı et al., 2021). Thioredoxin-1 (Trx-1), a redox regulating protein, inhibits ferroptosis in PD by positively regulating GSH and Gpx4 (Bai et al., 2021). Additionally, Shi’s moxa sticks effectively inhibit ferroptosis in rats with PD and improve the survival of dopaminergic neurons (Huang et al., 2021). Paeoniflorin, which is a water-soluble monoterpene glycoside extracted from the root of *Paeonia lactiflora* Pall, inhibits ferroptosis by activating the Akt/Nrf2/Gpx4 axis *in vitro* and exerts neuroprotective effects in PD (Wang L. et al., 2022).

Microglial activation and M1 polarization are related to Gpx4 and GSH content reduction and lipid peroxidation, all of which are involved in ferroptosis. Hou et al. (2019) revealed that neurodegeneration of the LC/NE system plays a critical role in mediating learning and memory dysfunction in a two pesticide-induced mouse model of PD and that this role was mediated through ferroptosis and microglia-mediated neuroinflammation.

### Potential therapeutic targets for Alzheimer’s disease (AD)

Alzheimer’s disease is characterized by synaptic loss and neuron death (Hambright et al., 2017). Ferroptosis may be an important mechanism underlying the neurodegeneration observed in diseases, such as AD, that are accompanied by Gpx4 depletion, lipid peroxide accumulation, and iron dyshomeostasis or improper iron transport mechanisms, which lead to the accumulation of this neurotoxic metal during hippocampal formation and in other cerebral areas (Hambright et al., 2017; Li L. B. et al., 2019).

The inhibition of ferroptosis in motor neurons is essential for the survival of these neurons, and this approach is one therapeutic strategy for treating AD and ALS. Martinez et al. (2019) reported that NSC-34 cells become more susceptible to Gpx4 deletion-induced ferroptosis when they differentiate into a more motor neuron-like state. These authors identified three key factors that affect motor neuron ferroptosis sensitivity: low serum antioxidant levels, inhibition of the *trans*-sulfuration pathway, and decreased Gpx4 levels (Martinez et al., 2019).

The AD is characterized by brain damage and neurotoxicity and is associated with oxidative stress, mitochondrial dysfunction, and impaired mitochondrial metabolism. NADPH oxidase 4 (NOX4), which is a major source of ROS, induces astrocyte ferroptosis accompanied by impaired mitochondrial metabolism by reducing five protein complexes in the astrocyte mitochondrial electron transport chain, NOX4 promotes oxidative stress-induced lipid peroxidation accompanied by the increased expression of the markers 4-HNE and MDA. Park et al. (2021) showed that NOX4 promotes the ferroptosis of astrocytes by triggering oxidative stress-induced lipid peroxidation via impaired mitochondrial metabolism in AD.

### Potential therapeutic targets for cerebral ischemia

Cerebral ischemia is characterized by ferroptosis. Carvacrol inhibits ferroptosis by increasing Gpx4 expression. In gerbils, carvacrol protects hippocampal neurons from ischemia-reperfusion injury by inhibiting ferroptosis by increasing Gpx4 expression (Guan et al., 2019). Melatonin treatment improves the learning and memory abilities of rats with hypoxic-ischemic brain damage through the AKT/Nrf2/Gpx4 axis (Gou et al., 2020). Naotaifang, a compound herbal preparation used in traditional Chinese medicine, also inhibits neuronal ferroptosis via the TFR1/DMT1 and SCL7A11/Gpx4 pathways in a rat model of acute cerebral ischemia (Lan et al., 2020). Exogenous NSCs inhibit ferroptosis through the p53/Gpx4/SLC7A11 pathway and exert neuroprotective effects on injured cells (Zhai et al., 2022).

### Potential therapeutic targets for intracerebral hemorrhage (ICH) and subarachnoid hemorrhage injury (SAH)

Various types of cell death, including necrosis, ferroptosis, and autophagy, are observed in the injured striatum during the acute phase of ICH. The pathological changes after ICH in mice, including abnormal synapses, degenerating neurons, and axonal demyelination, may provide new targets for ferroptosis that will be valuable for future studies on ICH pathology (Li et al., 2018).

The concentrations of Fe^2+^ and the expression of ferroptosis-related genes, including MDA, Cox-2, PTGS2, and RPL8, are increased during ICH (Chen et al., 2019). Peroxisome proliferator-activated receptor γ is a ligand-activated receptor that is a member of the nuclear hormone receptor family, and it synergistically interacts with the Nrf2 pathway to promote Gpx4 expression and inhibit ferroptosis (Duan et al., 2022). Crocin, which is a potential antioxidative agent, inhibits neuronal ferroptosis by increasing Fe^2+^ concentrations and Nrf2 (Abdalkader et al., 2018), Gpx4, ferritin heavy chain 1 (FTH1), and SLC7A11 expression, thus alleviating ICH (Wang F. et al., 2022). Additionally, ferroptosis is an essential form of OPC death that occurs during hemorrhagic stroke. Hemin-induced OPC death via decreased Gpx4 activity triggers lipid peroxide accumulation, and inhibiting ferroptosis rescues OPC death, attenuates IVH-induced WMI and promotes neurological function recovery (Shen et al., 2022).

Subarachnoid hemorrhage injury, characterized by inflammatory response and M1 microglial activation, is a devastating neurological emergency that accounts for high mortality and morbidity. 15-Lipoxygenase-1 (ALOX15), which is an inducer of ferroptosis, is overexpressed in microglia with Gpx4/GSH defects and abnormal mitochondria after SAH. Hemin probably induces ferroptosis in M2 microglia by upregulating ALOX15 and downregulating Gpx4 (Gao et al., 2022). L-*N*-(6)-iminoethyl-lysine (L-NIL), which is an inhibitor of iNOS, inhibits iNOS expression and promotes the ferroptosis of M1 microglia in SAH, and M1 microglial activation is considered to be related to the secretion of proinflammatory cytokines and neuroinflammation after SAH (Qu et al., 2022).

### Potential therapeutic targets for Pelizaeus–Merzbacher disease (PMD) and periventricular leukomalacia (PVL)

Pelizaeus–Merzbacher disease is caused by mutations in *proteolipid protein 1 (PLP1)*, resulting in early lethality and profound developmental delay. Oligodendrocytes expressing mutant *PLP1* exhibit key hallmarks of ferroptosis, including augmented lipid peroxidation, enhanced ROS production, abnormal iron metabolism, and hypersensitivity to free iron. Deferiprone (DFP), which is a small molecule iron chelator, decreases oligodendrocyte ferroptosis and facilitates oligodendrocyte differentiation and myelination in preclinical models of PMD *in vitro* and *in vivo* (Nobuta et al., 2019).

Periventricular leukomalacia is a syndrome that affects preterm infants and is caused primarily by the iron-dependent death of developing oligodendrocytes. Fer-1, SRS11-92, and Fer-1 analogs fully protected oligodendrocytes from cysteine deprivation-induced ferroptosis. Fer-1 prevents the depletion of cysteine and GSH by inhibiting their oxidative destruction (Skouta et al., 2014; Yang et al., 2014).

### Potential therapeutic targets for multiple sclerosis (MS) and experimental autoimmune encephalomyelitis (EAE)

Multiple sclerosis is a chronic demyelinating disease of the CNS. Oligodendrocyte loss, demyelination, and axon loss are the mechanisms underlying MS. Any factors that induce ferroptosis may accelerate the progression of MS, while agents that inhibit ferroptosis may reverse the progression of MS.

Iron that is released from oligodendrocytes during demyelination or iron that is derived from the breakdown products of hemoglobin is believed to amplify oxidative tissue injury in MS. Iron-dependent peroxidation by ROS and ferroptosis may participate in the demyelination and axon loss that occur during MS. Baldacchino et al. (2022) reported that hemin preferentially binds to myelin and axons to initiate a detrimental response, which results in targeted demyelination and axon loss in MS, and intracerebral degradation of hemoglobin may activate the progression of MS. Cuprizone, which is a copper chelator, disrupts the molecules that are involved in iron homeostasis and induces ferroptosis-induced oligodendrocyte loss and demyelination (Jhelum et al., 2020). In addition, ferroptosis, which is associated with miRNA-mediated inhibition of oligodendrocyte maturation, may be a principal target of MS and EAE (Ma et al., 2020).

The expression levels of Gpx4 and two other inhibitors of ferroptosis (γ-glutamylcysteine ligase and the cysteine/glutamate antiporter) are decreased in MS and its animal model EAE (Hu et al., 2019).

### Potential therapeutic targets for spinal cord injury (SCI)

Spinal cord injury is associated with motor neuronal ferroptosis and triggers ROS accumulation, while iron chelators, ROS inhibitors and ferroptosis inhibitors decrease iron overload-induced motor neuron ferroptosis and promote the recovery of motor function (Feng et al., 2021). Additionally, trehalose can effectively inhibit ferroptosis by activating the Nrf2/heme oxygenase-1 pathway (Gong et al., 2022). Recent studies have focused increasing attention on white matter injury and the probable underlying mechanism following SCI. DFO can repair SCI by inhibiting ferroptosis. Targeting ferroptosis is therefore a promising therapeutic approach for treating SCI (Yao et al., 2019). Sodium selenite, which is as an inhibitor of ferroptosis, promotes the neurological function of rats with SCI via the specificity protein 1/Gpx4 pathway (Chen et al., 2022). Lip-1 not only inhibits mitochondrial lipid peroxidation but also restores the levels of ferroptosis suppressor protein 1, GSH, and Gpx4 in oligodendrocytes (Fan et al., 2021). Fer-1, which is also an inhibitor of ferroptosis in oligodendrocytes, downregulates ferroptosis-related genes and the products of IREB2 and PTGS2, and can reduce iron and ROS accumulation, ultimately promoting neurological function recovery and reducing white matter injury after SCI in rats (Ge et al., 2021).

RSL-3, which is an inducer of oligodendrocyte ferroptosis, significantly increases the intracellular concentrations of ROS and MDA. In addition, it inhibits anti-ferroptosis pathways, such as the SLC7A11/GSH/Gpx4 pathway, and downregulates ACSL4 (Fan et al., 2021). Inhibiting system *Xc*^–^ may trigger ferroptosis in OLs. Ferroptosis is a major factor that contributes to glutamate-initiated oligodendrocyte death in TBI and stroke. Glutamate-induced ASM activation is associated with decreased GSH levels (Novgorodov et al., 2018).

The ferroptosis inhibitor, i.e., Fer-1, inhibits reactive astrocyte activation in an SCI model and may exert a therapeutic effect in other diseases of the CNS that are characterized by ferroptosis (Ge et al., 2021).

### Potential therapeutic targets for depression

Depression is considered to be caused by inflammation and oxidative stress. Jiao et al. (2021) revealed that ferroptosis activation might occur in the hippocampi of mice exposed to chronic unpredictable mild stress. Xiaoyaosan exerts antidepressant effects via the PEBP1-mediated inhibition of Gpx4 expression, thus inducing ferroptosis. Dang et al. (2022) analyzed hippocampal and medial prefrontal cortex tissues from rats. Edaravone, which is a free radical scavenger, exerts potent antidepressant, anxiolytic and neuroprotective effects via the Sirt1/Nrf2/heme oxygenase-1/Gpx4 signaling pathway, and Gpx4-mediated ferroptosis may modulate these effects. Ferroptosis may be a new target for future research on potent antidepressant therapy.

### Potential therapeutic targets for other CNS diseases

Amyotrophic lateral sclerosis is a motor neuron disease caused by the degeneration of motor neurons in the brain and spinal cord (Wang T. et al., 2022). Evans et al. (2022) reported that treating Gpx4NIKO mice with tamoxifen to ablate Gpx4 in neurons accelerates the progression of ALS caused by motor neuron ferroptosis. The MPO/HOCl pathway induces ferroptosis with lipid peroxidation by inhibiting Gpx4 and NADPH quinone oxidoreductase (NQO1) in mouse models of ALS (Peng et al., 2022). Experimental cerebral malaria may be caused by neuronal ferroptosis accompanied by upregulated TfR-1 and ACSL4 expression and downregulated Gpx4 expression. CD8(+) T cells may induce neuronal ferroptosis (Liang et al., 2022). Transferrin receptors are differentially expressed in normal human astrocytes and glioblastoma cells. Downregulated Gpx4 is accompanied by constant levels of cystine transporter and ACSL4 in glioblastoma, which suggests that dihydroartemisinin (DHA) induces ferroptosis Gpx4 under these conditions by directly targeting (Yi et al., 2020). Postoperative cognitive dysfunction is a common complication that occurs after surgery or anesthesia in elderly patients. Coimmunoprecipitation was used to observe the binding of mind bomb-2 (MIB2) to Gpx4. Zhao et al. (2021) found that sevoflurane anesthesia upregulated MIB2 in mouse hippocampal neurons. Depletion of MIB2 reduced the neuronal ferroptosis that is triggered by exposure to sevoflurane. Sepsis-associated encephalopathy, which is caused by an aberrant systemic immune response to infection, often manifests as severe diffuse cerebral dysfunction. Ferroptosis is involved in glutamate-induced excitotoxic neuronal death during the progression of sepsis-associated encephalopathy, and it is accompanied by Gpx4 depletion and transferrin and MDA upregulation (Xie et al., 2022).

Similar to liproxstatin-1, the function of melatonin, which is a ferroptosis inhibitor, is likely dependent on melatonin receptor 1B. Melatonin exerts cerebroprotective effects by inhibiting neuronal ferritin H-mediated ferroptosis after TBI (Rui et al., 2021).

Baicalein exerts protective effects against posttraumatic epileptic seizures by inhibiting ferroptosis, and 12/15-lipoxygenase (12/15-LOX) is likely to be involved in the neuroprotective effects of baicalein (Li Q. et al., 2019).

Central presbycusis is caused by auditory center degradation during aging, which is accompanied by the accumulation of iron and the upregulation of iron regulatory protein-2 (IRP-2) and TfR-1; these processes trigger ferroptosis by increasing the entry of iron into cells (Chen et al., 2020). Increased microglial expression of heme oxygenase-1 during aging and age-related neurodegenerative diseases has been correlated with the deposition of neurotoxic ferric iron. However, in aged mice exposed to lipopolysaccharide (LPS), major improvements in iron, inflammatory and behavioral alterations were observed when the mice were treated with the iron chelator DFO (Fernández-Mendívil et al., 2021).

Ferroptosis is associated with type 1 diabetes related cognitive dysfunction, and SLC40A1 mediates ferroptosis in type 1 diabetes (Hao et al., 2021).

Ferroptosis is involved in the progression of neuropathic pain in male rats by inhibiting astrocyte activation in the spinal dorsal horn. Wang et al. (2021) used a rat model of chronic contractile injury to mimic neuropathic pain. Chronic contractile injury caused mechanical and thermal stimulation of the injured hind paw, downregulated Gpx4, upregulated ACSL4, and increased the iron ion content in the spinal cords (Wang et al., 2021). Chronic brain injury in the cortex and hippocampus after TBI occurs almost exclusively in areas with iron deposits, while neuroprotection is accompanied by a reduction in astrocyte activation (Wehn et al., 2021).

## Conclusion and future directions

The goals of this review were to discuss the role of ferroptosis in the main cells of the CNS and to explore the potential targets of in different cells of the CNS for the treatment of pathological conditions. Multiple studies have reported that ferroptosis plays an important role in the progression of numerous CNS diseases including PD, AD, ALS, PMD, PVL, MS, EAE, TBI, and SCI. System *Xc*^–^ blockade, GSH depletion, Gpx4 inactivity, lipoxygenase activation, intracellular iron accumulation, and some related Nrf2 signaling pathways are the common ferroptotic mechanisms in CNS diseases. Some ferroptosis-related genes (GSH synthesis related genes, iron metabolism related genes, NADPH regeneration related genes, and lipid synthesis related genes) regulated by Nrf2 may be associated with the cell to cell interactions. Targeting pathways that regulate ferroptosis in central nervous cells is an emerging strategy for the treatment of neurological diseases. Some small molecules and traditional Chinese drugs that inhibit ferroptosis of glial cells, neurons, and pericytes are shown to be underlying therapeutic strategies for neurological diseases.

Many researchers have investigated neuronal ferroptosis in the CNS under pathological conditions; based on this review, we note that future studies are needed to clarify the association among glial cells, pericytes, and ferroptosis, which is also important for the development of therapeutic strategies for treating many pathological diseases. We believe that a better understanding of ferroptosis in various cells of the CNS will provide new opportunities for therapeutic intervention.

## Author contributions

YL, DX, and XW: conceptualization, software, validation, investigation, resources, writing—review and editing, and visualization. YL and DX: writing—original draft preparation. DX and XW: supervision. DX: funding acquisition. All authors read and approved the final manuscript.

## References

[B1] Abdalkader M. Lampinen R. Kanninen K. Malm T. Liddell J. (2018). Targeting Nrf2 to suppress ferroptosis and mitochondrial dysfunction in neurodegeneration. *Front. Neurosci.* 12:466. 10.3389/fnins.2018.00466 30042655 PMC6048292

[B2] Avcı B. Günaydın C. Güvenç T. Yavuz C. Kuruca N. Bilge S. (2021). Idebenone ameliorates rotenone-induced Parkinson’s disease in rats through decreasing lipid peroxidation. *Neurochem. Res.* 46 513–522. 10.1007/s11064-020-03186-w 33247801

[B3] Back S. A. Han B. H. Luo N. L. Chricton C. A. Xanthoudakis S. Tam J. (2002). Selective vulnerability of late oligodendrocyte progenitors to hypoxia-ischemia. *J. Neurosci.* 22 455–463. 11784790 10.1523/JNEUROSCI.22-02-00455.2002 PMC6758669

[B4] Back S. Rivkees S. (2004). Emerging concepts in periventricular white matter injury. *Semin. Perinatol.* 28 405–414. 15693397 10.1053/j.semperi.2004.10.010

[B5] Bai L. Yan F. Deng R. Gu R. Zhang X. Bai J. (2021). Thioredoxin-1 rescues MPP(+)/MPTP-induced ferroptosis by increasing glutathione peroxidase 4. *Mol. Neurobiol.* 58 3187–3197. 10.1007/s12035-021-02320-1 33634378

[B6] Bai Q. Liu J. Wang G. (2020). Ferroptosis, a regulated neuronal cell death type after intracerebral hemorrhage. *Front. Cell. Neurosci.* 14:591874. 10.3389/fncel.2020.591874 33304242 PMC7701249

[B7] Baldacchino K. Peveler W. J. Lemgruber L. Smith R. S. Scharler C. Hayden L. (2022). Myelinated axons are the primary target of hemin-mediated oxidative damage in a model of the central nervous system. *Exp. Neurol.* 354:114113. 10.1016/j.expneurol.2022.114113 35569511

[B8] Cao Y. Li Y. He C. Yan F. Li J. Xu H. (2021). Selective ferroptosis inhibitor liproxstatin-1 attenuates neurological deficits and neuroinflammation after subarachnoid hemorrhage. *Neurosci. Bull.* 37 535–549. 10.1007/s12264-020-00620-5 33421025 PMC8055759

[B9] Cardoso B. Hare D. Bush A. Roberts B. (2017). Glutathione peroxidase 4: A new player in neurodegeneration? *Mol. Psychiatry* 22 328–335. 10.1038/mp.2016.196 27777421

[B10] Chen B. Chen Z. Liu M. Gao X. Cheng Y. Wei Y. (2019). Inhibition of neuronal ferroptosis in the acute phase of intracerebral hemorrhage shows long-term cerebroprotective effects. *Brain Res. Bull.* 153 122–132. 10.1016/j.brainresbull.2019.08.013 31442590

[B11] Chen L. Hambright W. Na R. Ran Q. (2015). Ablation of the Ferroptosis inhibitor glutathione peroxidase 4 in neurons results in rapid motor neuron degeneration and paralysis. *J. Biol. Chem.* 290 28097–28106. 10.1074/jbc.M115.680090 26400084 PMC4653669

[B12] Chen X. Li D. Sun H. Wang W. Wu H. Kong W. (2020). Relieving ferroptosis may partially reverse neurodegeneration of the auditory cortex. *FEBS J.* 287 4747–4766. 10.1111/febs.15266 32112499

[B13] Chen Y. Zuliyaer T. Liu B. Guo S. Yang D. Gao F. (2022). Sodium selenite promotes neurological function recovery after spinal cord injury by inhibiting ferroptosis. *Neural Regen. Res.* 17 2702–2709. 10.4103/1673-5374.339491 35662217 PMC9165358

[B14] Codazzi F. Pelizzoni I. Zacchetti D. Grohovaz F. (2015). Iron entry in neurons and astrocytes: A link with synaptic activity. *Front. Mol. Neurosci.* 8:18. 10.3389/fnmol.2015.00018 26089776 PMC4452822

[B15] Cui Y. Zhang Y. Zhao X. Shao L. Liu G. Sun C. (2021a). ACSL4 exacerbates ischemic stroke by promoting ferroptosis-induced brain injury and neuroinflammation. *Brain Behav. Immun.* 93 312–321. 10.1016/j.bbi.2021.01.003 33444733

[B16] Cui Y. Zhang Z. Zhou X. Zhao Z. Zhao R. Xu X. (2021b). Microglia and macrophage exhibit attenuated inflammatory response and ferroptosis resistance after RSL3 stimulation via increasing Nrf2 expression. *J. Neuroinflammation* 18:249. 10.1186/s12974-021-02231-x 34717678 PMC8557003

[B17] Cui Z. Zhong Z. Yang Y. Wang B. Sun Y. Sun Q. (2016). Ferrous iron induces Nrf2 expression in mouse brain astrocytes to prevent neurotoxicity. *J. Biochem. Mol. Toxicol.* 30 396–403. 10.1002/jbt.21803 27037625

[B18] Czepiel M. Boddeke E. Copray S. (2015). Human oligodendrocytes in remyelination research. *Glia* 63 513–530. 10.1002/glia.22769 25421998

[B19] Dang R. Wang M. Li X. Wang H. Liu L. Wu Q. (2022). Edaravone ameliorates depressive and anxiety-like behaviors via Sirt1/Nrf2/HO-1/Gpx4 pathway. *J. Neuroinflammation* 19:41. 10.1186/s12974-022-02400-6 35130906 PMC8822843

[B20] Devos D. Cabantchik Z. I. Moreau C. Danel V. Mahoney-Sanchez L. Bouchaoui H. (2020). Conservative iron chelation for neurodegenerative diseases such as Parkinson’s disease and amyotrophic lateral sclerosis. *J. Neural Transm. (Vienna)* 127 189–203. 10.1007/s00702-019-02138-1 31912279

[B21] Dixon S. J. Lemberg K. M. Lamprecht M. R. Skouta R. Zaitsev E. M. Gleason C. E. (2012). Ferroptosis: An iron-dependent form of nonapoptotic cell death. *Cell* 149 1060–1072. 10.1016/j.cell.2012.03.042 22632970 PMC3367386

[B22] Domingues H. Portugal C. Socodato R. Relvas J. (2016). Oligodendrocyte, astrocyte, and microglia crosstalk in myelin development, damage, and repair. *Front. Cell Dev. Biol.* 4:71. 10.3389/fcell.2016.00071 27551677 PMC4923166

[B23] Duan C. Jiao D. Wang H. Wu Q. Men W. Yan H. (2022). Activation of the PPARγ prevents ferroptosis-induced neuronal loss in response to intracerebral hemorrhage through synergistic actions with the Nrf2. *Front. Pharmacol.* 13:869300. 10.3389/fphar.2022.869300 35517804 PMC9065416

[B24] Evans R. Chen L. Na R. Yoo K. Ran Q. (2022). The Gpx4NIKO mouse is a versatile model for testing interventions targeting ferroptotic cell death of spinal motor neurons. *Neurotox. Res.* 40 373–383. 10.1007/s12640-021-00469-0 35043381 PMC9035057

[B25] Fan B. Pang Y. Li W. Zhao C. Zhang Y. Wang X. (2021). Liproxstatin-1 is an effective inhibitor of oligodendrocyte ferroptosis induced by inhibition of glutathione peroxidase 4. *Neural Regen. Res.* 16 561–566. 10.4103/1673-5374.293157 32985488 PMC7996026

[B26] Feng Z. Min L. Chen H. Deng W. Tan M. Liu H. (2021). Iron overload in the motor cortex induces neuronal ferroptosis following spinal cord injury. *Redox Biol.* 43:101984. 10.1016/j.redox.2021.101984 33933882 PMC8105676

[B27] Fernández-Mendívil C. Luengo E. Trigo-Alonso P. García-Magro N. Negredo P. López M. (2021). Protective role of microglial HO-1 blockade in aging: Implication of iron metabolism. *Redox Biol.* 38:101789. 10.1016/j.redox.2020.101789 33212416 PMC7680814

[B28] Gao S. Zhou L. Lu J. Fang Y. Wu H. Xu W. (2022). Cepharanthine attenuates early brain injury after subarachnoid hemorrhage in mice via inhibiting 15-lipoxygenase-1-mediated microglia and endothelial cell ferroptosis. *Oxid. Med. Cell. Long.* 2022:4295208. 10.1155/2022/4295208 35186185 PMC8850040

[B29] Ge H. Xue X. Xian J. Yuan L. Wang L. Zou Y. (2021). Ferrostatin-1 alleviates white matter injury via decreasing ferroptosis following spinal cord injury. *Mol. Neurobiol.* 59 161–176. 10.1007/s12035-021-02571-y 34635980

[B30] Gong F. Ge T. Liu J. Xiao J. Wu X. Wang H. (2022). Trehalose inhibits ferroptosis via NRF2/HO-1 pathway and promotes functional recovery in mice with spinal cord injury. *Aging* 14 3216–3232. 10.18632/aging.204009 35400664 PMC9037257

[B31] Gou Z. Su X. Hu X. Zhou Y. Huang L. Fan Y. (2020). Melatonin improves hypoxic-ischemic brain damage through the Akt/Nrf2/Gpx4 signaling pathway. *Brain Res. Bull.* 163 40–48. 10.1016/j.brainresbull.2020.07.011 32679060

[B32] Guan X. Li X. Yang X. Yan J. Shi P. Ba L. (2019). The neuroprotective effects of carvacrol on ischemia/reperfusion-induced hippocampal neuronal impairment by ferroptosis mitigation. *Life Sci.* 235:116795. 10.1016/j.lfs.2019.116795 31470002

[B33] Guiney S. J. Adlard P. A. Lei P. Mawal C. H. Bush A. I. Finkelstein D. I. (2020). Fibrillar α-synuclein toxicity depends on functional lysosomes. *J. Biol. Chem.* 295 17497–17513. 10.1074/jbc.RA120.013428 33453994 PMC7762966

[B34] Hambright W. Fonseca R. Chen L. Na R. Ran Q. (2017). Ablation of ferroptosis regulator glutathione peroxidase 4 in forebrain neurons promotes cognitive impairment and neurodegeneration. *Redox Biol.* 12 8–17. 10.1016/j.redox.2017.01.021 28212525 PMC5312549

[B35] Hao L. Mi J. Song L. Guo Y. Li Y. Yin Y. (2021). SLC40A1 mediates ferroptosis and cognitive dysfunction in type 1 diabetes. *Neuroscience* 463 216–226. 10.1016/j.neuroscience.2021.03.009 33727075

[B36] Hoshino T. Yamakado H. Takahashi R. Matsuzawa S. (2020). Susceptibility to erastin-induced ferroptosis decreases during maturation in a human oligodendrocyte cell line. *FEBS Open Bio.* 10 1758–1764. 10.1002/2211-5463.12923 32608563 PMC7459400

[B37] Hou L. Sun F. Sun W. Zhang L. Wang Q. (2019). Lesion of the locus coeruleus damages learning and memory performance in paraquat and maneb-induced mouse Parkinson’s disease model. *Neuroscience* 419 129–140. 10.1016/j.neuroscience.2019.09.006 31634513

[B38] Hu C. Nydes M. Shanley K. Morales Pantoja I. Howard T. Bizzozero O. (2019). Reduced expression of the ferroptosis inhibitor glutathione peroxidase-4 in multiple sclerosis and experimental autoimmune encephalomyelitis. *J. Neurochem.* 148 426–439. 10.1111/jnc.14604 30289974 PMC6347488

[B39] Huang L. Zhang Y. Zhao L. Chen Q. Li L. (2022). Ferrostatin-1 polarizes microglial cells toward M2 phenotype to alleviate inflammation after intracerebral hemorrhage. *Neurocrit. Care* 36 942–954. 10.1007/s12028-021-01401-2 35099711

[B40] Huang Z. Si W. Li X. Ye S. Liu X. Ji Y. (2021). Moxibustion protects dopaminergic neurons in Parkinson’s disease through antiferroptosis. *Evid. Based Complement. Alternat. Med.* 2021:6668249. 10.1155/2021/6668249 34122606 PMC8191581

[B41] Ishii T. Warabi E. Mann G. (2019). Circadian control of BDNF-mediated Nrf2 activation in astrocytes protects dopaminergic neurons from ferroptosis. *Free Radic. Biol. Med.* 133 169–178. 10.1016/j.freeradbiomed.2018.09.002 30189266

[B42] Jhelum P. Santos-Nogueira E. Teo W. Haumont A. Lenoël I. Stys P. K. (2020). Ferroptosis mediates cuprizone-induced loss of oligodendrocytes and demyelination. *J. Neurosci.* 40 9327–9341. 10.1523/jneurosci.1749-20.2020 33106352 PMC7687057

[B43] Ji R. Donnelly C. Nedergaard M. (2019). Astrocytes in chronic pain and itch. *Nat. Rev. Neurosci.* 20 667–685. 10.1038/s41583-019-0218-1 31537912 PMC6874831

[B44] Jiang X. Stockwell B. Conrad M. (2021). Ferroptosis: Mechanisms, biology and role in disease. *Nat. Rev. Mol. Cell Biol.* 22 266–282. 10.1038/s41580-020-00324-8 33495651 PMC8142022

[B45] Jiao H. Yang H. Yan Z. Chen J. Xu M. Jiang Y. (2021). Traditional Chinese formula Xiaoyaosan alleviates depressive-like behavior in CUMS mice by regulating PEBP1-GPX4-mediated ferroptosis in the hippocampus. *Neuropsychiatr. Dis. Treat.* 17 1001–1019. 10.2147/ndt.s302443 33854318 PMC8039849

[B46] Kapralov A. A. Yang Q. Dar H. H. Tyurina Y. Y. Anthonymuthu T. S. Kim R. (2020). Redox lipid reprogramming commands susceptibility of macrophages and microglia to ferroptotic death. *Nat. Chem. Biol.* 16 278–290. 10.1038/s41589-019-0462-8 32080625 PMC7233108

[B47] Keuters M. H. Keksa-Goldsteine V. Dhungana H. Huuskonen M. T. Pomeshchik Y. Savchenko E. (2021). An arylthiazyne derivative is a potent inhibitor of lipid peroxidation and ferroptosis providing neuroprotection in vitro and in vivo. *Sci. Rep.* 11:3518. 10.1038/s41598-021-81741-3 33568697 PMC7876050

[B48] Lan B. Ge J. Cheng S. Zheng X. Liao J. He C. (2020). Extract of naotaifang, a compound Chinese herbal medicine, protects neuron ferroptosis induced by acute cerebral ischemia in rats. *J. Integr. Med.* 18 344–350. 10.1016/j.joim.2020.01.008 32107172

[B49] Li J. Li M. Ge Y. Chen J. Ma J. Wang C. (2022). β-amyloid protein induces mitophagy-dependent ferroptosis through the CD36/PINK/PARKIN pathway leading to blood-brain barrier destruction in Alzheimer’s disease. *Cell Biosci.* 12:69. 10.1186/s13578-022-00807-5 35619150 PMC9134700

[B50] Li L. B. Chai R. Zhang S. Xu S. F. Zhang Y. H. Li H. L. (2019). Iron exposure and the cellular mechanisms linked to neuron degeneration in adult mice. *Cells* 8:198. 10.3390/cells8020198 30813496 PMC6406573

[B51] Li Q. Li Q. Jia J. Sun Q. Zhou H. Jin W. (2019). Baicalein exerts neuroprotective effects in FeCl(3)-induced posttraumatic epileptic seizures via suppressing ferroptosis. *Front. Pharmacol.* 10:638. 10.3389/fphar.2019.00638 31231224 PMC6568039

[B52] Li Q. Weiland A. Chen X. Lan X. Han X. Durham F. (2018). Ultrastructural characteristics of neuronal death and white matter injury in mouse brain tissues after intracerebral hemorrhage: Coexistence of ferroptosis, autophagy, and necrosis. *Front. Neurol.* 9:581. 10.3389/fneur.2018.00581 30065697 PMC6056664

[B53] Li S. Zhou C. Zhu Y. Chao Z. Sheng Z. Zhang Y. (2021). Ferrostatin-1 alleviates angiotensin II (Ang II)- induced inflammation and ferroptosis in astrocytes. *Int. Immunopharmacol.* 90:107179. 10.1016/j.intimp.2020.107179 33278745

[B54] Li X. Yang S. Li M. Li X. Tian Q. Xiao F. (2021). Formaldehyde induces ferroptosis in hippocampal neuronal cells by upregulation of the Warburg effect. *Toxicology* 448:152650. 10.1016/j.tox.2020.152650 33259821

[B55] Liang J. Shen Y. Wang Y. Huang Y. Wang J. Zhu Q. (2022). Ferroptosis participates in neuron damage in experimental cerebral malaria and is partially induced by activated CD8(+) T cells. *Mol. Brain.* 15:57. 10.1186/s13041-022-00942-7 35725567 PMC9208218

[B56] Liu Y. Wang Z. Cao C. Xu Z. Lu J. Shen H. (2022). Aquaporin 4 depolarization-enhanced transferrin infiltration leads to neuronal ferroptosis after subarachnoid hemorrhage in mice. *Oxid. Med. Cell. Longev.* 2022:8808677. 10.1155/2022/8808677 35761873 PMC9233479

[B57] Liu Z. Lv X. Song E. Song Y. (2020). Fostered Nrf2 expression antagonizes iron overload and glutathione depletion to promote resistance of neuron-like cells to ferroptosis. *Toxicol. Appl. Pharmacol.* 407:115241. 10.1016/j.taap.2020.115241 32937103

[B58] Liu Z. Lv X. Yang B. Qin Q. Song E. Song Y. (2021). Tetrachlorobenzoquinone exposure triggers ferroptosis contributing to its neurotoxicity. *Chemosphere* 264(Pt 1):128413. 10.1016/j.chemosphere.2020.128413 33017703

[B59] Ma Q. Matsunaga A. Ho B. Oksenberg J. Didonna A. (2020). Oligodendrocyte-specific argonaute profiling identifies microRNAs associated with experimental autoimmune encephalomyelitis. *J. Neuroinflammation* 17:297. 10.1186/s12974-020-01964-5 33046105 PMC7552381

[B60] Martinez A. Mirkovic J. Stanisz Z. Patwari F. Yang W. S. (2019). NSC-34 motor neuron-like cells are sensitized to ferroptosis upon differentiation. *FEBS Open Bio.* 9 582–593. 10.1002/2211-5463.12577 30984534 PMC6443867

[B61] Morris G. Berk M. Carvalho A. Maes M. Walker A. Puri B. (2018). Why should neuroscientists worry about iron? The emerging role of ferroptosis in the pathophysiology of neuroprogressive diseases. *Behav. Brain Res.* 341 154–175. 10.1016/j.bbr.2017.12.036 29289598

[B62] Nobuta H. Yang N. Ng Y. Marro S. Sabeur K. Chavali M. (2019). Oligodendrocyte death in pelizaeus-merzbacher disease is rescued by iron chelation. *Cell Stem Cell* 25 531–541.e6. 10.1016/j.stem.2019.09.003 31585094 PMC8282124

[B63] Novgorodov S. Voltin J. Gooz M. Li L. Lemasters J. Gudz T. (2018). Acid sphingomyelinase promotes mitochondrial dysfunction due to glutamate-induced regulated necrosis. *J. Lipid Res.* 59 312–329. 10.1194/jlr.M080374 29282302 PMC5794425

[B64] Park M. W. Cha H. W. Kim J. Kim J. H. Yang H. Yoon S. (2021). NOX4 promotes ferroptosis of astrocytes by oxidative stress-induced lipid peroxidation via the impairment of mitochondrial metabolism in Alzheimer’s diseases. *Redox Biol.* 41:101947. 10.1016/j.redox.2021.101947 33774476 PMC8027773

[B65] Peng J. Pan J. Mo J. Peng Y. M. P. O. (2022). /HOCl Facilitates apoptosis and ferroptosis in the SOD1(G93A) motor neuron of amyotrophic lateral sclerosis. *Oxid. Med. Cell. Long.* 2022:8217663. 10.1155/2022/8217663 35178161 PMC8845144

[B66] Qin X. Tang Q. Jiang X. Zhang J. Wang B. Liu X. (2020). Zinc oxide nanoparticles induce ferroptotic neuronal cell death in vitro and in vivo. *Int. J. Nanomed.* 15 5299–5315. 10.2147/ijn.S250367 32884256 PMC7436556

[B67] Qu W. Cheng Y. Peng W. Wu Y. Rui T. Luo C. (2022). Targeting iNOS Alleviates early brain injury after experimental subarachnoid hemorrhage via promoting ferroptosis of M1 Microglia and reducing neuroinflammation. *Mol. Neurobiol.* 59 3124–3139. 10.1007/s12035-022-02788-5 35262869

[B68] Rui T. Wang H. Li Q. Cheng Y. Gao Y. Fang X. (2021). Deletion of ferritin H in neurons counteracts the protective effect of melatonin against traumatic brain injury-induced ferroptosis. *J. Pineal Res.* 70:e12704. 10.1111/jpi.12704 33206394

[B69] Santambrogio P. Ripamonti M. Cozzi A. Raimondi M. Cavestro C. Meo I. D. (2022). Massive iron accumulation in PKAN-derived neurons and astrocytes: Light on the human pathological phenotype. *Cell Death Dis.* 13:185. 10.1038/s41419-022-04626-x 35217637 PMC8881507

[B70] Schriever S. C. Zimprich A. Pfuhlmann K. Baumann P. Giesert F. Klaus V. (2017). Alterations in neuronal control of body weight and anxiety behavior by glutathione peroxidase 4 deficiency. *Neuroscience* 357 241–254. 10.1016/j.neuroscience.2017.05.050 28627418

[B71] Segovia K. N. McClure M. Moravec M. Luo N. L. Wan Y. Gong X. (2008). Arrested oligodendrocyte lineage maturation in chronic perinatal white matter injury. *Ann. Neurol.* 63 520–530. 10.1002/ana.21359 18393269 PMC3140464

[B72] Shen D. Wu W. Liu J. Lan T. Xiao Z. Gai K. (2022). Ferroptosis in oligodendrocyte progenitor cells mediates white matter injury after hemorrhagic stroke. *Cell Death Dis.* 13:259. 10.1038/s41419-022-04712-0 35318305 PMC8941078

[B73] Skouta R. Dixon S. J. Wang J. Dunn D. E. Orman M. Shimada K. (2014). Ferrostatins inhibit oxidative lipid damage and cell death in diverse disease models. *J. Am. Chem. Soc.* 136 4551–4556. 10.1021/ja411006a 24592866 PMC3985476

[B74] Sofroniew M. (2020). Astrocyte reactivity: Subtypes, states, and functions in cns innate immunity. *Trends Immunol.* 41 758–770. 10.1016/j.it.2020.07.004 32819810 PMC7484257

[B75] Stockwell B. R. Angeli J. P. Bayir H. Bush A. I. Conrad M. Dixon S. J. (2017). Ferroptosis: A regulated cell death nexus linking metabolism, redox biology, and disease. *Cell* 171 273–285. 10.1016/j.cell.2017.09.021 28985560 PMC5685180

[B76] Su X. Huang L. Qu Y. Xiao D. Mu D. (2019). Pericytes in cerebrovascular diseases: An emerging therapeutic target. *Front. Cell. Neurosci.* 13:519. 10.3389/fncel.2019.00519 31824267 PMC6882740

[B77] Tang Q. Bai L. Zou Z. Meng P. Xia Y. Cheng S. (2018). Ferroptosis is newly characterized form of neuronal cell death in response to arsenite exposure. *Neurotoxicology* 67 27–36. 10.1016/j.neuro.2018.04.012 29678591

[B78] Urbán N. Blomfield I. Guillemot F. (2019). Quiescence of adult mammalian neural stem cells: A highly regulated rest. *Neuron* 104 834–848. 10.1016/j.neuron.2019.09.026 31805262

[B79] Wang F. Li W. Shen L. Jiang T. Xia J. You D. (2022). Crocin alleviates intracerebral hemorrhage-induced neuronal ferroptosis by facilitating Nrf2 nuclear translocation. *Neurotox. Res.* 40 596–604. 10.1007/s12640-022-00500-y 35380368

[B80] Wang H. Huo X. Han C. Ning J. Chen H. Li B. (2021). Ferroptosis is involved in the development of neuropathic pain and allodynia. *Mol Cell Biochem.* 476 3149–3161. 10.1007/s11010-021-04138-w 33864570

[B81] Wang L. An H. Yu F. Yang J. Ding H. Bao Y. (2022). The neuroprotective effects of paeoniflorin against MPP(+)-induced damage to dopaminergic neurons via the Akt/Nrf2/GPX4 pathway. *J. Chem. Neuroanat.* 122:102103. 10.1016/j.jchemneu.2022.102103 35489613

[B82] Wang T. Tomas D. Perera N. D. Cuic B. Luikinga S. Viden A. (2022). Ferroptosis mediates selective motor neuron death in amyotrophic lateral sclerosis. *Cell Death Dif.* 29 1187–1198. 10.1038/s41418-021-00910-z 34857917 PMC9177596

[B83] Wehn A. Khalin I. Duering M. Hellal F. Culmsee C. Vandenabeele P. (2021). RIPK1 or RIPK3 deletion prevents progressive neuronal cell death and improves memory function after traumatic brain injury. *Acta Neuropathol. Commun.* 9:138. 10.1186/s40478-021-01236-0 34404478 PMC8369637

[B84] Weiland A. Wang Y. Wu W. Lan X. Han X. Li Q. (2019). Ferroptosis and its role in diverse brain diseases. *Mol. Neurobiol.* 56 4880–4893. 10.1007/s12035-018-1403-3 30406908 PMC6506411

[B85] Wen L. Zhou T. Jiang Y. Gong L. Yang B. (2021). Identification of prenylated phenolics in mulberry leaf and their neuroprotective activity. *Phytomedicine* 90:153641. 10.1016/j.phymed.2021.153641 34281775

[B86] Wu C. Zhao W. Yu J. Li S. Lin L. Chen X. (2018). Induction of ferroptosis and mitochondrial dysfunction by oxidative stress in PC12 cells. *Sci. Rep.* 8:574. 10.1038/s41598-017-18935-1 29330409 PMC5766540

[B87] Wu J. Yang J. Cao Y. Li H. Zhao H. Yang S. (2020). Iron overload contributes to general anaesthesia-induced neurotoxicity and cognitive deficits. *J. Neuroinflammation* 17:110. 10.1186/s12974-020-01777-6 32276637 PMC7149901

[B88] Wu T. Liang X. Liu X. Li Y. Wang Y. Kong L. (2020). Induction of ferroptosis in response to graphene quantum dots through mitochondrial oxidative stress in microglia. *Part. Fibre Toxicol.* 17:30. 10.1186/s12989-020-00363-1 32652997 PMC7353734

[B89] Xiao D. Qu Y. Pan L. Li X. Mu D. (2018). MicroRNAs participate in the regulation of oligodendrocytes development in white matter injury. *Rev. Neurosci.* 29 151–160. 10.1515/revneuro-2017-0019 29120862

[B90] Xie Z. Xu M. Xie J. Liu T. Xu X. Gao W. (2022). Inhibition of ferroptosis attenuates glutamate excitotoxicity and nuclear autophagy in a CLP septic mouse model. *Shock* 57 694–702. 10.1097/shk.0000000000001893 35066511

[B91] Yang W. S. SriRamaratnam R. Welsch M. E. Shimada K. Skouta R. Viswanathan V. S. (2014). Regulation of ferroptotic cancer cell death by GPX4. *Cell* 156 317–331. 10.1016/j.cell.2013.12.010 24439385 PMC4076414

[B92] Yao J. Mu Y. Gage F. (2012). Neural stem cells: Mechanisms and modeling. *Protein Cell* 3 251–261. 10.1007/s13238-012-2033-6 22549585 PMC4875476

[B93] Yao X. Zhang Y. Hao J. Duan H. Zhao C. Sun C. (2019). Deferoxamine promotes recovery of traumatic spinal cord injury by inhibiting ferroptosis. *Neural Regen. Res.* 14 532–541. 10.4103/1673-5374.245480 30539824 PMC6334606

[B94] Yi R. Wang H. Deng C. Wang X. Yao L. Niu W. (2020). Dihydroartemisinin initiates ferroptosis in glioblastoma through GPX4 inhibition. *Biosci. Rep.* 40:BSR20193314. 10.1042/bsr20193314 32452511 PMC7313443

[B95] Yu L. Su X. Li S. Zhao F. Mu D. Qu Y. (2020). Microglia and their promising role in ischemic brain injuries: An update. *Front. Cell. Neurosci.* 14:211. 10.3389/fncel.2020.00211 32754016 PMC7365911

[B96] Yu S. Li Z. Zhang Q. Wang R. Zhao Z. Ding W. (2022). GPX4 degradation via chaperone-mediated autophagy contributes to antimony-triggered neuronal ferroptosis. *Ecotoxicol. Environ. Saf.* 234:113413. 10.1016/j.ecoenv.2022.113413 35305351

[B97] Zhai Q. Ren Y. Ni Q. Song Z. Ge K. Guo Y. (2022). Transplantation of human umbilical cord mesenchymal stem cells-derived neural stem cells pretreated with neuregulin1β ameliorate cerebral ischemic reperfusion injury in rats. *Biomolecules* 12:428. 10.3390/biom12030428 35327620 PMC8945978

[B98] Zhang Y. Sun C. Zhao C. Hao J. Zhang Y. Fan B. (2019). Ferroptosis inhibitor SRS 16-86 attenuates ferroptosis and promotes functional recovery in contusion spinal cord injury. *Brain Res.* 1706 48–57. 10.1016/j.brainres.2018.10.023 30352209

[B99] Zhao L. Gong H. Huang H. Tuerhong G. Xia H. (2021). Participation of mind bomb-2 in sevoflurane anesthesia induces cognitive impairment in aged mice via modulating ferroptosis. *ACS Chem. Neurosci.* 12 2399–2408. 10.1021/acschemneuro.1c00131 34121396

